# First use of a new extended reality tool for preoperative planning in coronary artery bypass surgery: a case-report

**DOI:** 10.1093/jscr/rjae383

**Published:** 2024-06-03

**Authors:** Sulayman el Mathari, Alexander Broersen, Jouke Dijkstra, Amir H Sadeghi, Bastiaan Grisèl, Robert A F de Lind van Wijngaarden, Robert J M Klautz, Jolanda Kluin

**Affiliations:** Department of Cardiothoracic Surgery, Amsterdam University Medical Center, 1105 AZ Amsterdam, The Netherlands; Department of Radiology, Leiden University Medical Center, 2333 ZA Leiden, The Netherlands; Department of Radiology, Leiden University Medical Center, 2333 ZA Leiden, The Netherlands; Department of Cardiothoracic Surgery, Erasmus University Medical Center, 3015 GD Rotterdam, The Netherlands; Creative Data Studio, 3029 AK Rotterdam, The Netherlands; Department of Cardiothoracic Surgery, Amsterdam University Medical Center, 1105 AZ Amsterdam, The Netherlands; Department of Cardiothoracic Surgery, Amsterdam University Medical Center, 1105 AZ Amsterdam, The Netherlands; Department of Cardiothoracic Surgery, Leiden University Medical Center, 2333 ZA Leiden, The Netherlands; Department of Cardiothoracic Surgery, Erasmus University Medical Center, 3015 GD Rotterdam, The Netherlands

**Keywords:** extended reality, preoperative planning, coronary artery bypass grafting, cardiac surgery

## Abstract

A 73-year-old male presented with angina symptoms and was diagnosed with three-vessel coronary artery disease by use of computed tomography angiography and coronary angiography. This diagnosis necessitated coronary artery bypass grafting (CABG) surgery. A custom made AI-driven algorithm was used to generate a patient-specific three-dimensional coronary artery model from computed tomography angiography imaging data. This framework enabled precise segmentation and reconstruction of the coronary vasculature, yielding an accurate anatomical and pathological representation. Subsequently, this generated model was integrated into a novel extended reality tool for preoperative planning and intraoperative guidance in CABG surgery. Both preoperatively and intraoperatively, the tool augmented spatial orientation and facilitated precise stenosis localization, thereby enhancing the surgeon’s operative proficiency. This case report underscores the utility of advanced extended reality tools in cardiovascular surgery, emphasizing their pivotal role in refining surgical planning and execution.

## Introduction

Coronary angiography (CAG) is the gold standard for preoperative planning in coronary artery bypass grafting (CABG) [1]. Despite its clinical significance, this invasive imaging modality contains limitations. Primarily, it relies on a two-dimensional representation of the intricate three-dimensional (3D) coronary anatomy and coronary analyses can be hard due to overlapping arteries. Additionally, objective disease quantification is lacking due to subjective eye-balling used for estimating stenosis severity, facilitating inter-observer disagreement. Furthermore, preoperative planning images are limited to those captured during CAG, restricting the available perspectives. And, technically, the method falls short in evaluating potential intramural courses of coronary arteries.

Recognizing these constraints, there is a need for an innovative tool to overcome these limitations. New imaging modalities, such as extended reality (XR), facilitate exploring less invasive and more effective techniques for preoperative planning in CABG [2]. An XR interactive preoperative planning tool rendering a full 3D view of the patients’ own coronary system could be a potential solution for the aforementioned limitations. Such a tool could not only enhance spatial orientation by presenting the 3D anatomy but also ensures a 360° perspective, allows simulation of different surgical strategies and enables objective quantification of stenosis through appropriate measuring techniques.

The authors engineered a custom XR preoperative planning tool to explore the potential advantages of such a tool. This state-of-the-art tool is able to generate patient-specific 3D coronary artery models by use of an artificial intelligence (AI) based algorithm and was used in a clinical case (Visual Abstract) described in this paper.

## Material and methods

### Segmentation and quantification of open lumen area

**Figure 1:**
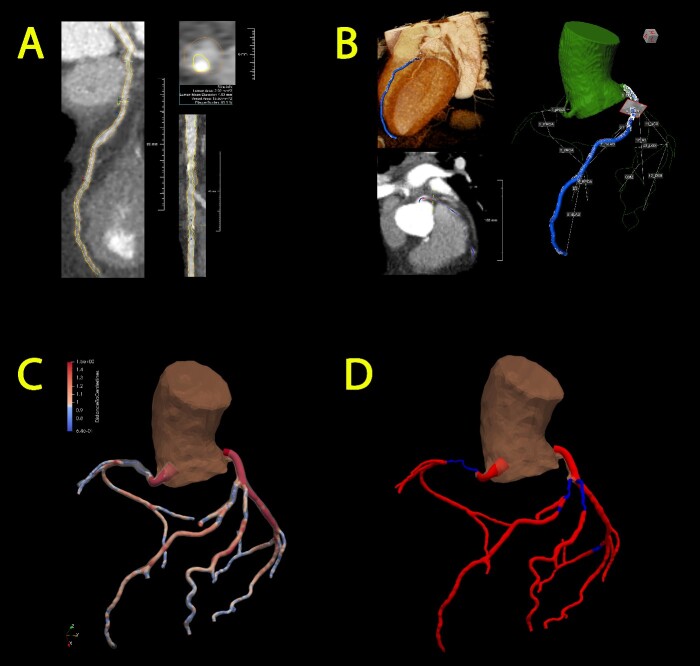
Semi-automated segmentation of the coronary artery tree, followed by manual refinement of each segmented artery (A) and transformation into a 3D representation (B). Subsequently, incorporation into an overall coronary artery structure follows through two models: the gradual flow model (C) and the stenosis detection model (D). Optimal blood flow is represented in red, while diminished blood flow is denoted by the color blue.

A custom AI-based algorithm was developed for semi-automated segmentation, which allows to create a patient-specific coronary artery model using computed tomography angiography (CTA) imaging. CTA data are imported into dedicated software QAngio CT Research Edition (v3.1.5.1 Medis Medical Imaging, Leiden, The Netherlands), where an initial segmentation of the coronary anatomy is generated. Subsequently, a manual review is conducted to refine the segmentation when necessary (Fig. 1A and B). Vessels with an outer diameter less than 1.5 mm are excluded from segmentation in consideration of their clinical irrelevance.

**Figure 2:**
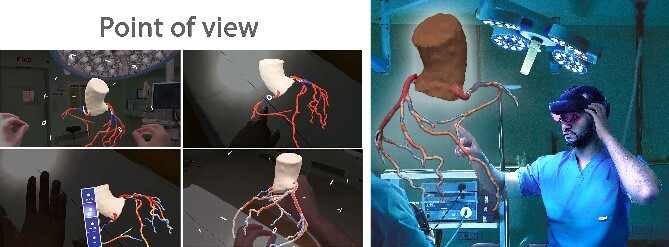
CoronaryXR platform. A user context is shown alongside a point of view of the system.

The model incorporates quantitative data pertaining to the lumen area, derived from measurements taken at varying distances from the open lumen wall to the centerline of the vessel at specific points. These data serve as a metric for assessing stenosis severity within the coronary artery model. Two distinct 3D models are constructed utilizing the acquired data; (1) a gradual flow model featuring a continuous representation of the lumen area variations (Fig. 1C) and (2) a stenosis detection model that identifies transition points where the area undergoes a 70% reduction or increase (Fig. 1D), thereby pinpointing the initiation and conclusion of significant stenotic vessel segments.

### Extended reality platform CoronaryXR

The generated models are loaded into a custom-made mixed reality platform named CoronaryXR, working on the Microsoft Hololens 2 device (Fig. 2). This XR environment facilitates immersive interaction and exploration of coronary anatomy, offering 360° visualization, segment marking, quantified stenosis assessment and graft simulations. Video 1 demonstrates a point-of-view user experience.

## Case report

**Figure 3:**
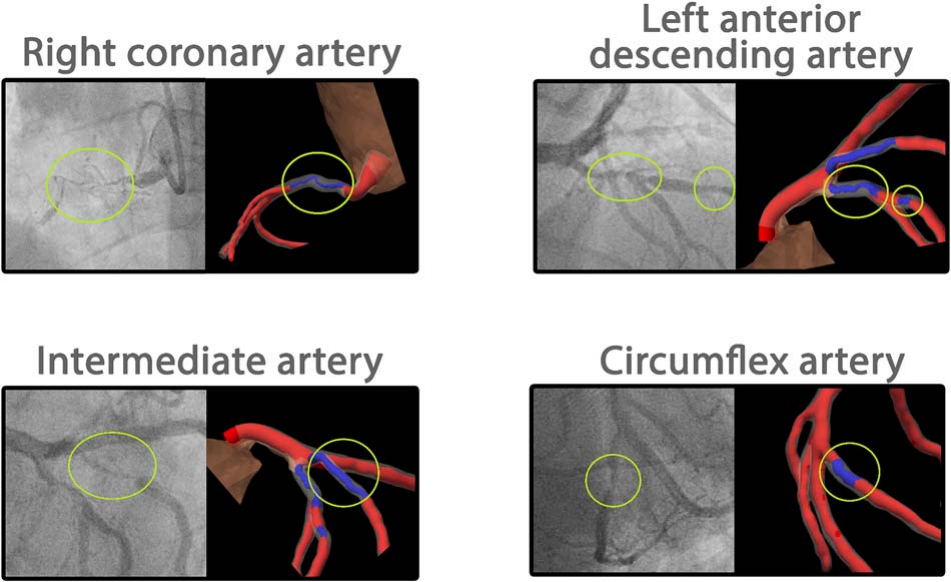
Coronary angiogram results of the patient aligned with the 3D coronary artery model on the CoronaryXR platform. Yellow circles highlight a total occlusion in the right coronary artery and significant stenosis in the left anterior descending, intermediate and circumflex arteries.

A 73-year-old male presented with angina symptoms, prompting a diagnostic evaluation with CTA. Results of the CTA scan raised suspicion of three-vessel coronary artery disease. Subsequent confirmation was sought through CAG, revealing a total occlusion in the right coronary artery (RCA) and significant stenosis in the left anterior descending (LAD), circumflex (Cx) and intermediate (IM) coronary arteries (Fig. 3). Simultaneously, CTA imaging data was used to generate a 3D segmentation of the coronary arteries with quantification of stenosis and showed the same lesions as on CAG. This segmentation was loaded on the CoronaryXR platform and facilitated a comprehensive 360° assessment, quantification of stenosis severity and simulation of the impending surgical intervention. The assessing surgeon used this tool in the preoperative phase and reported enhanced spatial orientation and acknowledged the tool’s value in quantifying the stenosis severity, along with the possibility to conduct a virtual simulation considering different grafting configurations before the surgery took place in reality. Notably, the tool was perceived as user-friendly, contributing to a positive user experience. The patient underwent CABG, during which an arterial graft (left internal mammary artery) was placed on the LAD, complemented by a saphenous vein graft on the IM, Cx and RCA. There were no complications in the postoperative setting and the patient was discharged 6 days after surgery.

## Discussion

Multiple applications of XR have been established in various surgical domains for preoperative planning. However, its preoperative utilization in cardiac surgery remains relatively constrained. Building on the success of 3D printed models that demonstrated the enhanced value of 3D visualization in congenital surgery [3], integrating XR introduces a more sustainable, time-efficient and cost-effective dimension to this domain. This case report presents a proof-of-concept through a clinical case, showcasing the feasibility and efficacy of an XR tool in the preoperative planning and intraoperative guidance of CABG surgery by enhancing the surgeon’s experience both before and during surgery.

This innovative approach not only enhances surgeons’ spatial orientation concerning surgical targets but also allows for quantitative disease assessment. Furthermore, it facilitates the exploration of diverse surgical strategies within a digital simulation environment, presenting opportunities to optimize surgical outcomes and refine patient care protocols in cardiac surgery. However, despite its promising potential, there is a need for future validation studies to assess both the added value of XR in routine clinical practice and the accuracy of disease quantification facilitated by the segmentation protocol.

Nonetheless, the integration of XR into preoperative planning and intraoperative guidance represents advancement for CABG procedures, particularly in an era witnessing a rise in minimally invasive techniques.

## Supplementary Material

Video_1_rjae383

Visual_Abstract_rjae383
