# Supplementary material for: First use of a new extended reality tool for preoperative planning in coronary artery bypass surgery: a case-report
Source: J Surg Case Rep. 2024 Jun 3;2024(6):rjae383. doi: 10.1093/jscr/rjae383 (PMC11146210; doi:10.1093/jscr/rjae383)

First use of a new extended reality tool for preoperative planning in coronary artery bypass surgery: a case-report

CASE REPORT

73-year old male with a total occlusion of the RCA and significant lesions in the LAD, Cx and IM

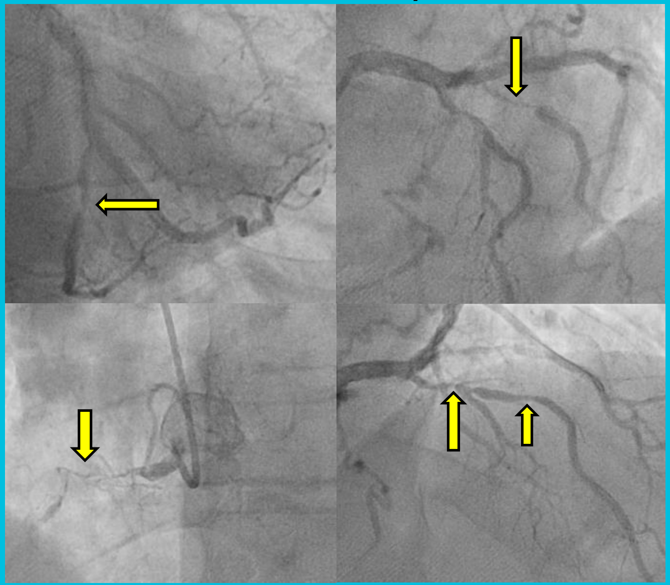

INNOVATION

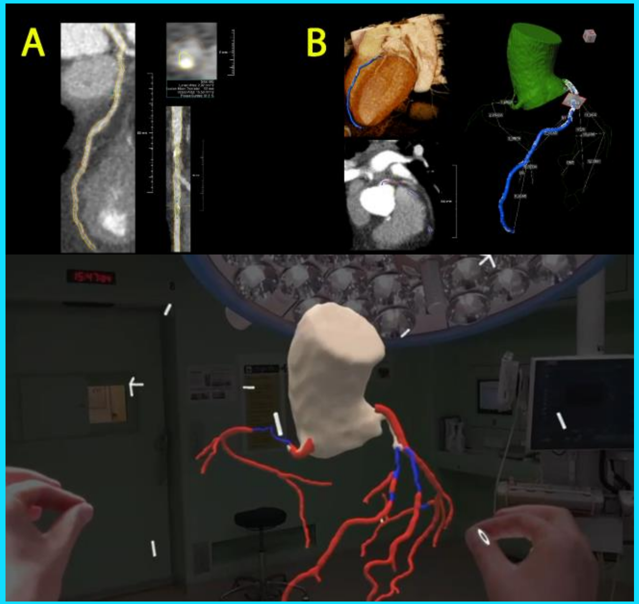

AI-based 3D segmentation of coronary anatomy and visualization in XR for preoperative planning

RESULT

XR coronary anatomy from preoperative planning used during surgery for intraoperative guidance

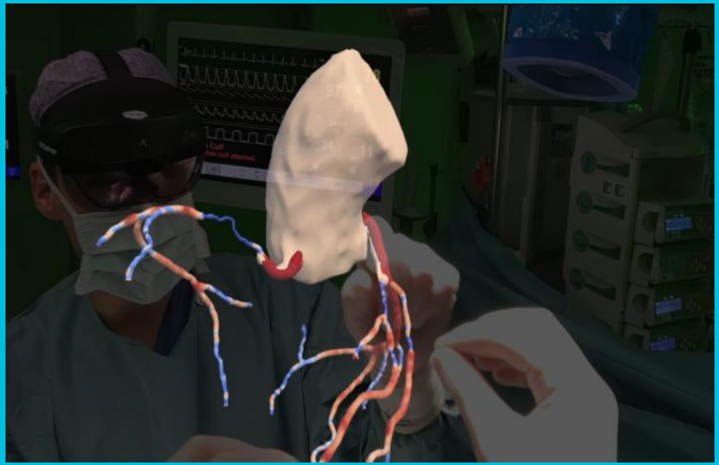

Supplement: Visual_Abstract_rjae383 [file visual_abstract_rjae383.pdf]
